# Exosomes and microvesicles in normal physiology, pathophysiology, and renal diseases

**DOI:** 10.1007/s00467-017-3816-z

**Published:** 2017-11-27

**Authors:** Anne-lie Ståhl, Karl Johansson, Maria Mossberg, Robin Kahn, Diana Karpman

**Affiliations:** Department of Pediatrics, Clinical Sciences Lund, Lund University, 22185 Lund, Sweden

**Keywords:** Extracellular vesicles, Exosomes, Microvesicles, Kidney, Inflammation, Thrombosis

## Abstract

Extracellular vesicles are cell-derived membrane particles ranging from 30 to 5,000 nm in size, including exosomes, microvesicles, and apoptotic bodies. They are released under physiological conditions, but also upon cellular activation, senescence, and apoptosis. They play an important role in intercellular communication. Their release may also maintain cellular integrity by ridding the cell of damaging substances. This review describes the biogenesis, uptake, and detection of extracellular vesicles in addition to the impact that they have on recipient cells, focusing on mechanisms important in the pathophysiology of kidney diseases, such as thrombosis, angiogenesis, tissue regeneration, immune modulation, and inflammation. In kidney diseases, extracellular vesicles may be utilized as biomarkers, as they are detected in both blood and urine. Furthermore, they may contribute to the pathophysiology of renal disease while also having beneficial effects associated with tissue repair. Because of their role in the promotion of thrombosis, inflammation, and immune-mediated disease, they could be the target of drug therapy, whereas their favorable effects could be utilized therapeutically in acute and chronic kidney injury.

## Introduction

Intercellular communication is essential for multicellular organisms and cells communicate by a variety of mechanisms such as direct cell–cell contact, transfer of secreted molecules or intercellular transfer of extracellular vesicles (EVs). EVs are membrane-bound vesicles released by cells under physiological and pathological conditions. As EVs circulate in the blood, they may act as shuttle vectors or signal transducers both locally and at a distance from their site of origin [1]. Another function of EVs is the removal of unwanted molecular material or cellular waste [2], conceivably as a means of maintaining cellular integrity.

Extracellular vesicles are subdivided into exosomes, microvesicles, and apoptotic bodies (Table 1). Exosomes are the smallest vesicles (30–100 nm) released by the fusion of multivesicular bodies containing intraluminal vesicles with the plasma membrane. Microvesicles are vesicular structures (0.1–1.0 μm) shed by outward blebbing of the plasma membrane. The largest EVs (1–5 μm) are apoptotic bodies that are formed during the late stages of apoptosis [5, 10]. These subtypes of extracellular vesicles differ in their mechanism of biogenesis, as described below. This review focuses mainly on exosomes and microvesicles. Certain studies have not specifically analyzed the subtype of vesicle, in which case we refer to the general term EVs.

**Table 1:** Main characteristics of exosomes, microvesicles, and apoptotic bodies

	Exosomes	Microvesicles	Apoptotic bodies	References
Size	30–100 nm	100–1,000 nm	1–5 μm	[3]
Origin	Intraluminal vesicles within multivesicular bodies	Plasma membrane and cellular content	Plasma membrane, cellular fragments	[4]
Mechanism of formation	Fusion of multivesicular bodies with the plasma membrane	Outward blebbing of the plasma membrane	Cell shrinkage and programmed cell death	[5, 6]
Release	Constitutive and/or cellular activation	Constitutive and/or cellular activation	Apoptosis	[4]
Time of release	Ten minutes or more	Few seconds	–	[7, 8]
Pathways	ESCRT-dependent Tetraspanin-dependent Ceramide-dependent Stimuli-dependent	Ca^2+^-dependent Stimuli- and cell-dependent	Apoptosis-related	[3]
Lipid membrane composition	Enriched in cholesterol and ceramide, expose phosphatidylserine, contain lipid rafts	Expose phosphatidylserine, enriched in cholesterol and diacylglycerol, contain lipid rafts	–	[3, 9]
Content	Proteins, mRNA, miRNA, lipids	Proteins, mRNA, miRNA, lipids	Cell organelles, proteins, nuclear fractions, DNA, coding and noncoding RNA, lipids	[3]

*ESCRT* endosomal sorting complex required for transport

Although microvesicles and exosomes are structurally similar, they differ in size, lipid composition, content, and cellular origin (Table 1). EVs may be shed, under physiological or pathological conditions, into the extracellular environment either constitutively or upon activation, hypoxia, oxidative stress, senescence or apoptosis [4]. The release of vesicles may be induced by the stimulation of purinergic receptors [11], by shear stress or apoptosis [12, 13] and by proinflammatory mediators [14] or thrombin [15]. In addition, bacterial virulence factors, such as Shiga toxin and lipopolysaccharides [16] and uremic toxins [17] induce the release of EVs.

Microvesicles carry membrane-derived receptors, proteins, including cytokines, chemokines, proteins involved in cellular signaling and/or migration, lipids, carbohydrates, and genetic material including mRNA and microRNAs (miRNAs) [4]. Their contents depend on the parent cell, the microenvironment and on the triggers preceding their release [5, 18–21]. The transfer of these substances to recipient cells may affect the phenotype of the target cell. EVs transport combinations of multiple mediators and are therefore considered a more powerful means of intercellular communication than the transfer of single molecules. Circulating microvesicles are mainly of platelet, erythrocyte, leukocyte, and endothelial origin [22–25]. Urinary microvesicles originate mainly from podocytes, tubular cells, and epithelial cells lining the urogenital tract [2].

## Extracellular vesicle biogenesis and release

**Fig. 1:**
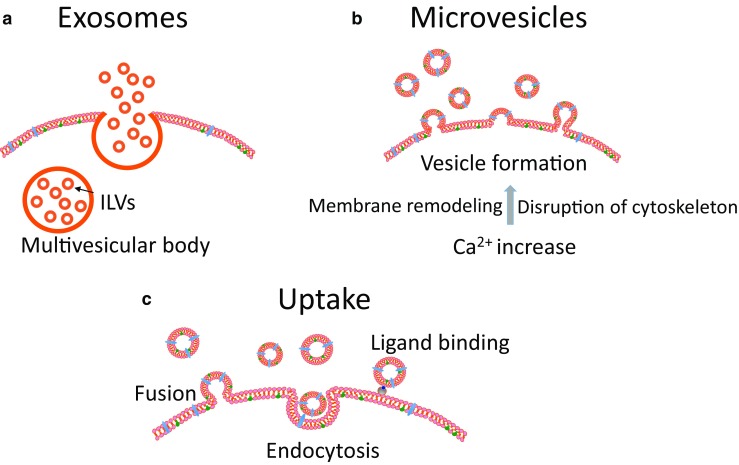
Schematic presentation of the release and uptake of extracellular vesicles. **a** Exosomes are released from late endosomes termed multivesicular bodies bearing intraluminal vesicles (*ILVs*) intracellularly. When the multivesicular bodies fuse with the plasma membrane and empty their contents, ILVs are released and are termed exosomes once they are extracellular. Exosomes are the smallest extracellular vesicles (Table 1). **b** Microvesicles are shed directly from the plasma membrane, thereby carrying membrane markers of the parent cell. Microvesicle formation is calcium-dependent and associated with loss of membrane asymmetry and disruption of the cellular cytoskeleton. **c** Extracellular vesicle uptake by target cells may occur via fusion of the vesicle membrane with the cell membrane or by endocytosis. The vesicle may also transduce an intracellular signal by ligand binding to a receptor on the recipient cell

Exosomes are the product of the fusion of a subset of late endosomes, called multivesicular bodies, with the plasma membrane releasing their contents including intraluminal vesicles (ILVs). Once extracellular, these vesicles are termed exosomes (Fig. 1) [3]. ILV formation is regulated via the endosomal sorting complex required for transport (ESCRT, four protein complexes that guide intracellular cargo) [26], and/or by non-ESCRT-related mechanisms, including tetraspanins [27] and membrane lipids [28].

Microvesicles are released from cells under physiological conditions, especially during cell growth [29]. Microvesicle shedding is increased when the cells are activated owing to cell injury, proinflammatory stimulants, hypoxia, oxidative stress or shear stress [30, 31]. Microvesicles are formed by outward protrusion or budding of the plasma membrane. This process is initiated by an increase in intracellular cytosolic calcium that activates calpain, a calcium-sensitive protease that detaches membrane proteins from the intracellular cytoskeleton [32], and gelsolin bound to actin filaments [33]. This leads to remodeling of the cytoskeleton, by cleaving the actin protein network, enabling blebbing to occur. Microvesicles are shed from plasma membrane micro-domains known as lipid rafts or caveolae domains [34]. The plasma membrane is composed of a lipid bilayer in which phosphatidylserine is located in the inner leaflet of the resting cell. The enzymes flippase, floppase, and scramblase control phospholipid asymmetry [35]. When the cell is activated, increased cytosolic calcium activates floppase (allowing lipid movement to the outer membrane) and scramblase (enabling bi-directional lipid movement), whereas flippase (allowing lipid movement to the inner membrane) is inactivated, resulting in flopping of negatively charged phosphatidylserine to the outer leaflet of the phospholipid bilayer [20]. This process does not always occur, as some microvesicles do not expose phosphatidylserine on their outer leaflet (Fig. 1) [36]. The presence of phosphatidylserine on the outer leaflet is readily detected, as it binds annexin V.

Microvesicles may express a slightly different repertoire of surface receptors or cytoplasmic components compared with the parent cell owing to a selective process during shedding [34]. Similarly, microvesicles released from activated cells do not express the same surface receptors as microvesicles shed during apoptosis [19] or from resting cells. This was demonstrated in vasculitis patients, microvesicles in patient samples taken during the active phase exhibited more CD62E and CD62P than those taken during remission and control samples [37].

## Clearance and uptake of extracellular vesicles

The quantity of EVs in the circulation reflects a balance between their generation and clearance. Microvesicles released into the circulation have a half-life of a couple of minutes to a few hours [38], during which they may be taken up by neighboring or distant cells. In humans undergoing cardiopulmonary bypass, transfusion or dobutamine-induced cardiac stress, elevated levels of circulating microvesicles were detected, but these returned to baseline levels within 15 min to ~6 h [39, 40]. Interestingly, clearance of microvesicles may depend on the cellular origin of the microvesicles as platelet-derived microvesicles were cleared sooner than microvesicles released from red blood cells [40].

There are various mechanisms for the cellular uptake of vesicles depending on the cargo of the vesicle, intercellular communication (e.g. receptor-ligand interactions) and the microenvironment of the cell. The most common mechanism is endocytosis, whereby the extracellular vesicle is engulfed by the recipient cell [38]. There are several mechanisms of endocytosis, such as clathrin-dependent or -independent, caveolin-mediated, macropinocytosis, phagocytosis and lipid raft-mediated [41]. Uptake of EVs seems to depend on the type of recipient cell, its physiological state, and recognition of ligands or receptors on the recipient cell and EVs [41]. For example, vesicles shed from platelets interact with monocytes [42] and endothelial cells [22], but not with neutrophils [42]. Likewise, exosomes exposing the tetraspanin–integrin complex were selectively taken up by endothelial and pancreatic cells [43].

Another mechanism for microvesicle uptake is fusion, whereby the microvesicles fuse with the membranes of the recipient cell and the content of the vesicle is released into the cell. Platelets expressing P-selectin fuse with tissue-factor-rich monocyte-derived microvesicles, increasing the procoagulability of platelets [34]. Fusion efficiency is enhanced in an acidic microenvironment (Fig. 1) [44].

## Detection

Extracellular vesicles are mostly detected in blood samples, but also in cerebrospinal fluid [45], urine [46], synovial fluid [47], bronchoalveolar lavage fluid [48], breast milk [49], bile [50], saliva [51], and uterine fluid [52], and the findings may reflect a process occurring on their cells of origin. Techniques for extracellular vesicle detection are listed in Table 2 and briefly described below. Given the heterogeneity of EVs the detection methods vary depending on which vesicle population is studied. The small size of exosomes demands a high sensitivity analysis method, including nanoparticle tracking analysis and electron microscopy [54, 57]. For the detection of microvesicles flow cytometry is the most common technique.

**Table 2:** Methods for the detection of extracellular vesicles and their contents

Method	Detection limit	Quantitative	Qualitative	Advantages	Limitations	Applicable to	Reference
Flow cytometry	300 nm	Yes	Yes	Easily available, single particle counting, offers multi-antibody labeling of vesicles	Requires skilled staff, swarm detection, limitations in sizing of microvesicles, can miss small vesicles	Exosomes^a^, microvesicles and apoptotic bodies	[53]
Nanoparticle tracking analysis	50 nm	Yes^b^	Yes	Short sample preparation, high resolution, and size determination of vesicles	Limited use of fluorescence, photo-bleaching^c^, can miss larger vesicles	Exosomes and microvesicles	[54]
Dynamic light scattering	5 nm	No	No	Size determination of vesicles and good reproducibility	Does not measure morphology or composition of vesicles	Exosomes, microvesicles, and apoptotic bodies	[55]
Resistive pulse sensing	70 nm	Yes	No	Is semi-quantitative and enables single vesicle detection	Does not measure morphology or composition of vesicles, risk of pore clogging	Exosomes, microvesicles, and apoptotic bodies	[56]
Transmission electron microscopy	~1 nm	No	Yes	Multiple antibody labeling, high resolution, and structural analysis	Labor-intensive, requires extensive sample preparation and skilled staff, morphological changes of vesicles during sample preparation	Exosomes, microvesicles, and apoptotic bodies	[57]
Atomic force microscopy	<1 nm	No	No	Relative size distribution of the vesicles, structural analysis, and high resolution	Extensive sample preparation, morphological changes of vesicles during sample preparation	Exosomes, microvesicles, and apoptotic bodies	[58, 59]
Immunoblot	N/A	No	Yes	Vesicle content detection	Requires larger quantities. Does not distinguish between exosomes, microvesicles or soluble antigens	Exosomes, microvesicles, and apoptotic bodies	[60]
ELISA	N/A	No	Yes	Vesicle content detection and quantification	Can only measure captured vesicles, and requires larger quantities. Does not distinguish between exosomes, microvesicles or soluble antigens	Exosomes, microvesicles, and apoptotic bodies	[61]
Proteomics	N/A	No	Yes	Quantifiable proteomic analysis of vesicle content	Does not distinguish between exosomes, microvesicles or soluble antigens. Time-consuming	Exosomes, microvesicles, and apoptotic bodies	[62]

*N/A* not applicable

^a^Owing to limitations in detectable size, analysis of exosomes by flow cytometry requires conjugation to beads with a bound specific antibody and can thus not be quantified or detect other exosomes not binding the antibody [63]

^b^Nanoparticle tracking analysis can be used for the quantification of small vesicles such as exosomes, but not for larger vesicles [54]

^c^Photo-bleaching is the process by which a fluorescent antibody fades rapidly

### Flow cytometry

The flow cytometer detects microvesicles as small as 150 nm in diameter (depending on the sensitivity of the instrument). The principle of detection is based on vesicles passing through a laser beam. Modern flow cytometers may have many lasers and fluorescence detectors, which allow for labeling with multiple conjugated antibodies in the same sample [64]. Microvesicles may have phosphatidylserine on their outer membrane enabling the use of conjugated annexin V for their detection [65].

Although flow cytometry is widely used to detect microvesicles, it has some limitations. Flow cytometry does not detect the smallest microvesicles as individual events. Multiple microvesicles may be detected collectively as a single event, a phenomenon termed swarm detection (Table 2) [66]. In addition, small microvesicles may have a limited number of antibody binding sites, sterically restricting staining with multiple antibodies [65]. Thus, both the number of small microvesicles and their surface expression may be underestimated.

### Transmission electron microscopy

The transmission electron microscope (TEM) visualizes small structures (limited to approximately 1 nm) because of the high resolution of the technique. Immune electron microscopy entails adding a conjugated antibody to detect a specific antigen in the sample [67]. Negative staining is performed when the surrounding medium is stained, leaving the vesicles unstained and the contrast clearly visualizes the vesicles.

### Nanoparticle tracking analysis

Nanoparticle tracking analysis (NTA) examines EVs in the liquid phase using a laser beam that determines the size and concentration by filming the light scattering when the particles move under Brownian motion [54]. The technique detects vesicles with a size of 0.05–1 μm (modern instruments may lower the detection limit even further). NTA can be used in fluorescent mode, thus detecting labeled vesicles [54]. NTA with fluorescent mode provides both quantitative and qualitative information on the vesicles in suspension.

## Extracellular vesicles in physiological and pathological processes

During physiological and pathological processes, EVs are released and partake in cellular communication affecting processes such as coagulation and thrombosis, angiogenesis, immune modulation and inflammation, which are discussed in the following sections.

### Intercellular communication

Extracellular vesicles use various mechanisms to transfer information to recipient cells. They may bind to receptors on target cells, thereby transducing a signal, or transfer functional receptors, proteins, lipids, mRNA or miRNA from parent cells to recipient cells in which they may induce phenotypic changes.

#### Extracellular vesicles in cell signaling

Extracellular vesicles expose numerous signaling proteins and lipids on their surface and may thus bind to and stimulate target cells directly. For example, microvesicles from platelets exposing P-selectin were shown to bind to P-selectin glycoprotein ligand-1 (PSGL-1) on the surface of leukocytes, leading to leukocyte accumulation and aggregation [68]. During morphogenesis of multicellular organisms, shed microvesicles exposing the morphogen protein “wingless” bind to a family of G protein-coupled receptors called frizzled, thereby forming a gradient necessary for adequate tissue development [69]. Similarly, lymphocyte-derived microvesicles carrying the morphogen “hedgehog” may bind to its receptor on early hematopoietic stem cells and thereby induce differentiation into megakaryocytes [70].

#### Transfer of receptors

Extracellular vesicles can transfer functional receptors to target cells, allowing cell signaling in cells that originally lacked the receptor or enhancing the number of receptors. For example, microvesicles exposing the kinin B1 receptor transferred a functional receptor to endothelial cells and to human embryonic kidney cells [71]. The transfer of adhesion molecules and receptors from platelets to hematopoietic or malignant cells via platelet-derived microvesicles modulated their adhesion capacity and engraftment [72, 73]. Furthermore, microvesicles released from aggressive glioma cells transferred the oncogenic epidermal growth factor receptor (EGFR) to tumor cells causing a propagation of oncogenic activity [74]. The C-C chemokine receptor type 5 (CCR5) and C-X-C chemokine receptor type 4 (CXCR4) are important for HIV-1 uptake by cells. Microvesicle-mediated transfer of CCR5 and CXCR4 enabled HIV-1 to be internalized in cells previously not susceptible to the virus [75, 76], suggesting that this might be a means of disseminating HIV infection.

#### Transfer of proteins and lipids

Extracellular vesicles transport proteins such as cytokines, chemokines, and growth factors to neighboring or distant cells, resulting in modulation of the target cell. In addition, EVs may transfer functional channels. Exosomes originating in murine kidney-collecting duct cells (mCCDC11) transfer functional aquaporin 2 (AQP2), increasing water transport in recipient cells [77] and can thus potentially be involved in intra-renal signaling downstream in the nephron. Upon release, EVs may shelter proteins that would otherwise be phagocytosed or neutralized in free form in plasma, thus protecting their content from the host response [22]. This mode of transport can also be utilized by bacterial and viral components to evade the host response [22, 78]. Bioactive lipids, such as sphingosine 1-phosphate and arachidonic acid, are also transported within microvesicles [79]. Lipids in platelet microvesicles can increase adhesion between endothelial cells and monocytes [80]; hence, microvesicles not only affect recipient cells, but also other cells in their microenvironment.

#### Transfer of mRNA and microRNA

Extracellular vesicles are enriched in mRNA and miRNA, which can be transferred horizontally to and translated in recipient cells, thereby changing the phenotype of the cell. For example, microvesicles shed by endothelial progenitor cells induced activation of quiescent endothelial cells and stimulated angiogenesis by transfer of mRNA [81]. Mesenchymal stem cell (MSC)-derived EVs transfer mRNAs, inducing transcription and proliferation of tubular epithelial cells after in vivo injury [82]. Exosomes may regulate mRNA levels in recipient cells by delivering functional miRNA, thus blocking translation [83–85]. Transfer of miRNA by urinary exosomes to tubular cells modulated their function, as exemplified by diminished ROMK1 potassium channel levels in human collecting duct cells [86]. Exosomal transfer of certain miRNAs between immune cells conferred both a proinflammatory and an anti-inflammatory effect in vitro and in mice following endotoxin administration [87]. Likewise, vesicles derived from endothelial progenitor cells contain mRNAs coding for inhibitors of the complement system and anti-apoptotic molecules, thereby inhibiting complement-induced apoptosis and complement deposition on mesangial cells [88]. Interestingly, horizontal transfer of genetic material and the changes seen in the target cells were even demonstrated between cells of different species [89].

### Protection against stress and cell death

To what extent EVs contribute to homeostasis and cell survival by ridding cells of unwanted substances is unknown, but may explain why cells release vesicles into their surroundings. The presence of complement C5b-9 on shed microvesicles may preserve the integrity of the parent cells by elimination of complement and the risk of cytolysis [90]. EVs from healthy individuals contain active caspase-3 that was not found in the parental cells, suggesting that caspase-3 might have been removed from the cells to ensure survival [91]. Inhibition of microvesicle release from viable endothelial cells containing active caspase-3 triggered both apoptosis and detachment of the cells [92].

Intriguingly, dying cells release microvesicles bearing the adaptor protein Crkl during the early stages of apoptosis induced by the caspase 3 cascade. These microvesicles were isolated from glomeruli after injury and were shown to induce compensatory proliferation signaling in recipient cells [93, 94]. Taken together, release of microvesicles may rid the cell of toxic substances, but may also induce repair in neighboring cells.

### Coagulation and thrombosis

Extracellular vesicles play an important role in coagulation, platelet aggregation, and thrombosis. Pro-thrombotic properties of microvesicles are primarily associated with exposure of negatively charged phosphatidylserine and tissue factor [95]. Phosphatidylserine on circulating platelet- and monocyte-derived microvesicles provides binding sites for the assembly of coagulation factors such as factor IXa, Va, Xa, and VIII followed by thrombin generation [96]. Phosphatidylserine is also present on exosomal membranes [9]. It not only facilitates formation of coagulation complexes, but also promotes tissue factor activity [97]. Tissue factor is normally encrypted, but may be exposed on microvesicles released from platelets, monocytes or endothelial cells [16, 34, 98] and form a complex with factor VII/VIIa, thereby activating the extrinsic pathway of coagulation.

Platelet-derived microvesicles have a significantly higher pro-coagulant activity compared with activated platelets most probably because of their higher surface density of phosphatidylserine, factor Xa, P-selectin, and αIIbβ_3_ (glycoprotein IIb/IIIa) [99]. At the site of vascular injury, platelet-derived microvesicles support thrombus formation by facilitating the adhesion of platelets to endothelial cell matrix components [100]. The interaction between PSGL-1 on monocyte-derived microvesicles and selectins on platelets, endothelial cells or their shed microvesicles provides a basis for thrombus formation [34].

### Angiogenesis

Microvesicles derived from blood and endothelial and tumor cells [101] may possess angiogenic properties, as previously reviewed [102]. The angiogenic effect may be associated with exposure of surface molecules or growth factors within the vesicles. Lymphoid microvesicles induced production of endothelial nitric oxide formation, expression of adhesion molecules, in addition to in vitro angiogenesis and in vivo neovascularization in endothelial cells [103]. Endothelial cell-derived microvesicles induced invasion of endothelial cells into basement membranes followed by capillary-like structure formation in vitro [104]. These properties may be of importance during tissue injury, post-ischemic revascularization and regeneration [105], and thus have importance during acute kidney injury (AKI).

### Immune modulation

Extracellular vesicles play an important role in promoting immune responses, affecting both innate and adaptive immunity. Dendritic cell-derived exosomes enhanced the cytotoxic activity of natural killer cells [106]. Moreover, dendritic cell microvesicles stimulated epithelial cells to release pro-inflammatory cytokines [107], leukocyte-derived microvesicles activated the endothelium, upregulating adhesion molecules and releasing cytokines, leading to leukocyte recruitment [108] and platelet microvesicles affected the adhesion of monocytes to the endothelium [80].

Extracellular vesicles may have antigen-presenting properties, exposing major histocompatibility complexes (MHCs). Dendritic cells stimulated with lipopolysaccharide shed vesicles exposing MHC II, CD83, and the co-stimulatory molecule CD40 on their surface initiating a pro-inflammatory response in epithelial cells and T-cell activation [107, 109]. Interestingly, dendritic microvesicles containing tumor necrosis factor-α could initiate an innate immune response in epithelial cells, leading to cytokine release without transfer of antigen-presenting properties [107]. Microvesicles may also affect adaptive immunity, as platelet-derived microvesicles can increase immunoglobulin production by B-cells [110].

Activation of the complement system is usually directed against foreign antigens such as bacteria or damaged host cells. Complement activation and deposition of the membrane attack complex on blood cells is followed by the release of complement-coated microvesicles [111, 112]. Microvesicles bearing C1q reflect activation of the classical pathway of complement on the parent cell [113], whereas the presence of C3 reflects amplification of all three pathways of the complement via the alternative pathway [111]. Direct activation on vesicles, after shedding, may potentially also occur. Blood cell-derived EVs expose complement regulators on their surface such as complement receptor type 1 (C1R), membrane cofactor protein (CD46), decay accelerating factor (DAF/CD55) or CD59 [111, 114], thereby inhibiting assembly of the membrane attack complex (C5b-9) and preventing excessive complement activation. In addition, EVs opsonized by C3b are rapidly cleared from the circulation by phagocytes [115].

### Malignancies

Tumor cells release significant numbers of EVs [116] that may influence proliferation, migration, invasion, and immune escape of cancer cells as well as angiogenesis [117] and the tumor environment [118]. EVs may also prime distant organs to a pre-metastatic niche facilitating survival and growth of metastasis [119]. An important step in tumor development is inhibition of immune surveillance. Tumor-derived exosomes can suppress T-cell immunity [120], thereby contributing to tumor progression by modulating and preventing anti-tumor immune reactions. The topic of EVs in malignancies has been reviewed elsewhere [116, 121].

### Inflammation

Extracellular vesicles are capable of inducing both inflammatory and anti-inflammatory responses. This may be associated with the transfer of pro- and anti-inflammatory mediators and by inducing the release of cytokines from target cells [108, 122–124]. Both leukocyte- and platelet-derived microvesicles induced cytokine release from endothelial cells [122, 125], suggesting that microvesicles might participate in vascular damage and inflammatory disorders. Moreover, EVs may induce chemotaxis. Platelet-derived microvesicles stimulated recruitment of hematopoietic cells [73] and promoted leukocyte migration [126]. Glomerular endothelial cell-derived microvesicles exposing the kinin B1 receptor and interleukin 8 (IL-8) on their surface attracted neutrophils [127, 128]. Proximal tubular cells cultured in the presence of fenoldopam (a dopamine receptor agonist) released exosomes that reduced the production of reactive oxygen species in distal tubule and collecting duct cells [129], indicating the transfer of an anti-inflammatory response.

### Anti-microbial effects

Neutrophil-derived microvesicles have been demonstrated to possess antimicrobial properties with a bacteriostatic effect on the uropathogen *Escherichia coli* [130]. Urinary exosomes also possess antimicrobial peptides, inhibiting the growth of *E. coli* and inducing bacteriolysis [131]. It has also been postulated that tissue factor-bearing microvesicles may prevent bacteria in the urinary tract from spreading beyond the uroepithelial barrier [132].

## Extracellular vesicles as biomarkers and promoters of kidney disease

The prothrombotic, proinflammatory, and immunomodulatory properties associated with EVs, described above, may all contribute to and maintain tissue damage in the kidney and urinary tract during the development of AKI, glomerular and tubular diseases, infections, and chronic renal failure in addition to numerous other conditions affecting the kidney. These aspects have been comprehensively reviewed recently by our group and others [2, 128, 133]. Studies on the role of EVs in AKI have mostly been carried out in patients with sepsis, burns or other forms of acute tubular injury [134, 135]. Our group has focused on the role of microvesicles in hemolytic uremic syndrome and vasculitis, which will be elaborated on below. In Table 3, we summarize various renal conditions in which EVs have been described as biomarkers of disease, in blood or urine, and describe which characteristics contribute to the induction and propagation of tissue injury.

**Table 3:** Extracellular vesicles in renal diseases and diseases with renal involvement

Renal disease		Presence in bodily fluid or tissue	Type of extracellular vesicle	Cell of origin	Importance		References
					Biomarker	Association with pathophysiology or beneficial effect	
AKI/sepsis		Blood	EV^a^ or MV	P, E, L	+^b^		[136–142]
				ns		May induce proteinuria and renal failure	[134, 135, 143]
				P, E		Induce vessel reactivity	[138]
				E		Proadhesive (PECAM-1, endoglin-positive) in association with DIC	[139]
				E		Levels of EVs correlate inversely with survival	[140, 141]
				N		Antimicrobial properties	[130, 144]
		Bone marrow, blood, and tissue	Ex and MV	MSCs^c^ and renal progenitor cells		EVs have regenerative properties during AKI	[145–147]
		Urine	Ex or EV	ns	+^d^		[148–151]
CKD or ESRF		Blood	EV	P, L, RBC, E	+		[17, 152–156]
				P, E		Pro-thrombotic	[153, 154]
				E		Correlate with vascular dysfunction	[155, 157]
				E		Predict cardiovascular disease	[158]
		Urine	Ex		+^e^		[159, 160]
TMA	STEC-HUS	Blood	MV	P	C3 and C9^f^	Pro-thrombotic (TF- and PS-positive)	[16, 111]
				RBC		Partake in hemolysis	[23]
				P, N, M		Transfer Shiga toxin to the kidneys	[22]
	aHUS	Blood	MV	P		Pro-thrombotic (TF- and PS-positive)	[161]
	TTP	Blood	MV	P		Associated with calpain activity	[162]
				E	C3 and C9^f^	Pro-coagulant and proadhesive (VWF, CD62E, ICAM-1, PECAM-1, endoglin-positive)	[163, 164]
Vasculitis		Blood	MV	P, N, E	+^g^		[37, 165, 166]
				N		PS-, TF-, selectin-, integrin-, PR3- and MPO-positive	[167, 168]
				N or ns		Pro-thrombotic	[169, 170]
				N		Bind C1q^f^	[168]
				N		Activate endothelial cells and monocytes	[167]
		Blood and kidney		N		Transfer the kinin B1 receptor to endothelial cells inducing inflammation	[71]
		Blood		E		Induce neutrophil chemotaxis	[127]
IgA nephropathy		Urine	MV and Ex	RBC	+^h^		[171–174]
Nephrotic syndrome		Blood	EV	RBC, E, P		Pro-thrombotic (PS-positive)	[175]
			MV	E, M		May contribute to albuminuria^i^	
		Urine	MV and Ex	Pod	+^j^		[151, 176 –180]
Urinary tract infection/urosepsis		Blood	MV	ns		Pro-thrombotic (TF-positive)	[181]
		Urine	Ex			Antimicrobial properties	[131]
Tubulopathies	Bartter syndrome	Urine	Ex		+		[182, 183]
	Gitelman syndrome	Urine	Ex		+		[184]
	Diabetes insipidus	Urine	EV	ns		Aquaporin-2 and its response to vasopressin differ in NDI vs CDI	[185]
ADPKD		Urine	Ex		+^k^		[186]
						Inverse correlation of the polycystin-1 or polycystin-2/transmembrane 2 ratios with kidney volume	[187]
						Exosomes interacted with primary cilia of renal epithelial cells	[188]
Hypertension		Blood	EV	P, E	+^l^		[189]
			MV	E		Indicate vascular injury	[190]
			EV	E		Elevated in patients with microalbuminuria	[191]
		Urine	EV	ns	+		[182]
				Pod	+^m^		[192]
Renal transplantation		Blood	MV	P, L, RBC	+^n^	TF activity decreases after transplantation	[193]
			Ex			Antigen-presenting vesicles activate anti-donor T cells	[194]
			Ex	E		Transfer of CMV antigens	[195]
			MV	P, E		Treatment with ATG and calcineurin inhibitors induces the release of complement-coated MVs	[196, 197]
		Urine	MV	CD133+ nephron-derived	+	Delayed graft function and vascular injury	[198]
			Ex		+^o^		[199]
			Ex		+	NGAL marker of delayed graft function	[200, 201]
			Ex		+	Decreased aquaporin 1 indicative of ischemia–reperfusion injury	[202]
		Kidney	MV	P	+	Platelets and platelet-derived MVs at sites of endothelial damage	[203]
SLE		Blood	EV	E or ns	+^p^	Contribute to immune complex deposition and complement activation	[204, 205]
			MV	P		Prothrombotic	[206]
		Urine	Ex			miRNA 29 correlated inversely with renal fibrosis	[207]
APS		Blood	MV	E		Pro-thrombotic	[208, 209]
Atherosclerosis		Blood	MV	E, P, L		Pro-thrombotic and proinflammatory	[210]
Diabetes mellitus		Blood	EV	A, I, M	+	Beta cell metabolism, inflammation	[211]
			MV	P, E, L		Pro-thrombotic, proinflammatory, correlated with arterial stiffness	[212–215]
		Urine	Ex			Urinary exosomal regucalcin decreased in diabetic nephropathy	[216]
			Ex		+	mRNA and protein markers of diabetic nephropathy	[217, 218]

*AKI* acute kidney injury, *EV* extracellular vesicle, *MV* microvesicle, *P* platelet, *E* endothelial, *L* leukocyte, *PECAM-1* platelet endothelial cell adhesion molecule (CD31), *DIC* disseminated intravascular coagulation, *Ex* exosome, *ns* not specified (for exosomes the distinction of the parent cell is not possible unless a specified cell type was studied), *N* neutrophil, *MSC* mesenchymal stem cells, *M* monocyte, *RBC* red blood cell, *CKD* chronic kidney disease, *ESRF* end-stage renal failure, *TMA* thrombotic microangiopathy, *TF* tissue factor, *PS* phosphatidylserine, *PR3* proteinase 3, *MPO* myeloperoxidase. *STEC-HUS* Shiga toxin-producing *Escherichia coli*-hemolytic uremic syndrome, *aHUS* atypical HUS, *TTP* thrombotic thrombocytopenic purpura, *VWF* von Willebrand factor, *CD62E* E-selectin, *ICAM-1* intercellular adhesion molecule 1, *NDI* nephrogenic diabetes insipidus (DI), *CDI* central DI, *ADPKD* autosomal dominant polycystic kidney disease, *ATG* antithymocyte globulin, *SLE* systemic lupus erythematosus, *APS* anti-phospholipid syndrome, *A* adipocytes, *I* islet cells

^a^Detected extracellular vesicles were not specified as exosomes, microvesicles or apoptotic bodies

^b^Elevated extracellular vesicles and miRNA may serve as biomarkers

^c^The exosomal fraction is responsible for the regenerative effects [146]

^d^Na/H exchanger isoform 3, fetuin-A or activating transcription factor 3 may reflect tubular injury

^e^miRNA profiles correlated with perturbed renal function and renal fibrosis

^f^Indicating complement activation

^g^Correspond to the Birmingham vasculitis activity score

^h^A miRNA profile derived from miRNA containing microvesicles. Protein biomarkers include α1-antitrypsin, aminopeptidase N, vasorin precursor, ceruloplasmin, and podocalyxin

^i^In vitro incubation of microvesicles with podocytes

^j^Urinary extracellular vesicle fractions contain nephrin, transient receptor potential cation channel 6, inverted formin-2 and phospholipase A2 receptor and Wilms tumor-1. In membranous nephropathy the microvesicles were positive for Lysosome Membrane Protein 2

^k^A distinct miRNA profile

^l^Higher in severe hypertension compared to mild hypertension

^m^Associated with renovascular hypertension and lower estimated glomerular filtration rate

^n^Levels decrease after renal transplantation (less so in patients with cardiovascular disease) and correlate inversely with renal function

^o^A proteomics approach determined patterns of rejection

^p^Levels correlate with SLE activity score, glomerulonephritis, hypertension, previous arterial thrombosis, and lipidemia

### Microvesicles in hemolytic uremic syndrome

Circulating microvesicles are elevated in thrombotic microangiopathies. Microvesicles derived from platelets, neutrophils, monocytes, and red blood cells were detected in blood samples from patients with Shiga toxin-producing *E. coli* (STEC)-associated hemolytic uremic syndrome (HUS) [16, 22, 23, 111, 219]. Patients with thrombotic thrombocytopenic purpura (TTP) exhibit elevated levels of both platelet and endothelial-derived microvesicles, the latter coated with complement deposits [162, 163, 220].

Our studies have shown that circulating microvesicles in STEC-HUS are pro-thrombotic/procoagulant as they are both tissue factor- and phosphatidylserine-positive. These aspects could be reproduced in vitro when whole blood was stimulated with Shiga toxin and *E. coli* O157 lipopolysaccharide and shed pro-thrombotic microvesicles were mainly derived from platelets [16]. Similarly, platelet- and monocyte-derived microvesicles in patient samples and in in vitro toxin-stimulated samples were coated with deposits of C3 and C9, suggesting ongoing complement activation.

Patients with STEC-HUS also exhibited elevated C3 and C9 on microvesicles derived from red blood cells, and, interestingly, Shiga toxin could induce complement activation on red blood cells followed by hemolysis, thereby releasing microvesicles from red blood cells with deposits of the membrane attack complex C5b-9 [23].

Shiga toxin is transported in vivo bound to blood cells and after uptake in these cells released within microvesicles (reviewed in Karpman et al. [221]). Blood cell-derived microvesicles transport Shiga toxin to the kidney, where the toxin, within microvesicles, is taken up in glomerular endothelial cells and peritubular capillary endothelial cells. Within the endothelial cells, the microvesicles either empty their cargo or are transcytosed through the cells, and their corresponding basement membranes, into podocytes or tubular cells, respectively. Eventually the microvesicles empty their cargo, although the signal leading to this release of contents is unknown. Intracellular toxin undergoes retrograde transport, binds to ribosomes and induces cell death thus causing renal failure [22]. HUS is characterized by platelet activation and the formation of microthrombi, hemolysis, and acute renal failure. These studies show that microvesicles are not only biomarkers, but actively contribute to disease-specific processes during STEC-HUS by creating a pro-thrombotic environment, partaking in hemolysis, and transporting Shiga toxin into the kidney to induce renal cell death.

In similarity to the pro-thrombotic microvesicles demonstrated in patients with STEC-HUS, serum from patients with aHUS, with mutations in the complement regulator factor H, induced the release of tissue factor- and phosphatidylserine-positive platelet-derived microvesicles from normal washed platelets, effects that could be inhibited by the addition of normal factor H [161].

### Microvesicles in vasculitis and inflammatory disorders

Microvesicles shed from endothelial cells, platelets, and leukocytes were increased during the acute phase of vasculitis, returning to normal levels during remission [25, 37, 152]. Endothelial microvesicle levels in pediatric vasculitis correlated with the Birmingham Vasculitis Activity Score (BVAS), C-reactive protein, and erythrocyte sedimentation rate [165]. Likewise, endothelial microvesicles in adults with anti-neutrophil cytoplasmic antibodies (ANCA)-associated vasculitis (AAV) correlated with the BVAS [37] and could thus be used as a biomarker for disease activity [37, 165].

The ANCAs circulating in patients with AAV activated neutrophils, causing them to release microvesicles [167]. In patients with vasculitis, neutrophil microvesicles activated endothelial cells, leading to the release of cytokines [108, 122, 167]. Neutrophil microvesicles may expose proteinase 3 (PR3) and myeloperoxidase (MPO) on their surfaces enabling ANCA to bind. Microvesicles were pro-thrombotic as they promoted the generation of thrombin [167], and could thus contribute to the thromboembolic complications seen in vasculitis.

Our studies have demonstrated systemic activation of the kinin system in children and adults with vasculitis underlying the profound vascular inflammation [222, 223]. We have shown that neutrophil-derived microvesicles bearing the kinin B1 receptor, expressed on cells during chronic inflammation, can transfer the receptor to cells lacking the receptor (demonstrated using transfected and wild-type HEK cells) and to glomerular endothelial cells, thereby promoting the inflammatory response. The phenomenon was confirmed in kidney biopsies showing that B1-receptor-positive neutrophil-derived microvesicles dock on glomerular endothelial cells in vivo during vasculitis [71]. Furthermore, during extensive vascular injury, endothelial microvesicles are released, also bearing the B1 receptor [127]. The B1-receptor-positive endothelial microvesicles recruited neutrophils, thus enhancing the inflammation. Interestingly, C1 inhibitor, the main inhibitor of the kinin system, inhibited the release of the chemotactic glomerular endothelial microvesicles.

In systemic lupus erythematosus (SLE), platelet-derived microvesicles are significantly increased and correlate with thrombin generation, suggesting a role in the thromboembolic state [206]. Other aspects, such as the contribution to immune complex deposition, are presented in Table 3.

Antiphospholipid syndrome is an autoimmune disease associated with antiphospholipid antibodies and thrombotic complications. Patients with antiphospholipid syndrome have elevated endothelial and platelet-derived microvesicles compared with controls and the endothelial vesicles may be pro-thrombotic [208].

## The effect of renal replacement therapy and drugs on extracellular vesicles

Treatments given during acute and chronic renal failure may affect levels of EVs. Dialysis treatment (hemodialysis and peritoneal dialysis) not only does not remove EVs, it may increase levels in comparison with healthy controls and after treatment sessions [17, 153]. The same is true for miRNA levels, which do not decrease after hemodialysis [136]. Treatment with recombinant erythropoietin may enhance levels of platelet-derived microvesicles, whereas the presence of an arteriovenous fistula has no effect on microvesicle levels [154].

To our knowledge, the effect of plasma exchange on levels of EVs has not been specifically addressed, but plasma exchange should presumably remove EVs. This has been suggested in the treatment of patients with SLE and antiphospholipid syndrome [224].

Various drugs used in the treatment of renal disease, including anti-hypertensive medications such as calcium channel blockers, amiloride, and beta blockers, or statins, may affect the release of EVs, as reviewed [128, 225]. Amiloride affects both the release and uptake of vesicles [226, 227].

## The renal regenerative capacity of extracellular vesicles

Mesenchymal stem cells and endothelial progenitor cells secrete EVs that have been demonstrated to induce nephron regeneration and repair by inhibiting apoptosis and promoting tubular proliferation. These effects have been documented in vitro [228] and in vivo [229] and are attributed to the transfer of both growth factors and RNAs (mRNAs and miRNAs) [230]. As described above, EVs can stimulate angiogenesis, and transfer growth factors such as vascular endothelial growth factor, hepatocyte growth factor [231], insulin-like growth factor-1 (IGF-1), adrenomedullin, and stromal cell-derived factor-1 (SDF1) [4]. Horizontal transfer of the IGF-1 receptor mRNA transcript via MSC EVs to damaged tubular cells induced proliferation [232]. EVs derived from MSCs localize to the kidney [233] and have been extensively investigated, in preclinical studies, for their therapeutic potential to protect tubuli and repair ischemia/reperfusion-induced injury [234].

## Extracellular vesicles as vehicles for drug delivery

The capacity of EVs to deliver proteins, lipids, and nucleic acids to recipient cells has therapeutic potential. EVs can be designed to target specific recipient cells. Cells can be genetically altered to express ligands on their membrane that are also present on EVs released from the cells. These ligands can bind to receptors on the target cell [235]. Thus, EVs can be loaded with therapeutic substances for delivery to target cells. These exciting developments in EV-based therapeutics may be used in future clinical trials and have been recently reviewed [236, 237].

## Conclusions

Extracellular vesicles play an important role in normal intercellular communication. They can be detected as biomarkers of disease owing to their excessive numbers and their properties and may also contribute to the development of diseases, including kidney disease, by inducing inflammation, vascular injury, and thrombosis in addition to modulating the immune response. Their contribution to the induction and progression of renal diseases may lead to the development of treatments geared toward temporary reduction of EVs systemically in the circulation, or locally in the kidney and urinary tract. Treatments that reduce the release or uptake of EVs need to take into account the notion that EVs may also be cytoprotective, as their release and the removal of unwanted or damaging substances from their parent cells may maintain cellular integrity. EVs may have potentially beneficial properties associated with tubular regeneration and the induction of angiogenesis. The therapeutic potential and nephroprotective effects of EVs, owing to their capacity to shuttle proteins, lipids, and genetic cargo to recipient cells, are being explored in preclinical studies, which may lead to clinical trials in the future.
